# 3-Bromo-9-(4-chloro­benz­yl)-9*H*-carbazole

**DOI:** 10.1107/S1600536809019448

**Published:** 2009-06-06

**Authors:** Zi-Dan Xiao, Tong Pan, Dao-Wu Yang

**Affiliations:** a College of Chemistry and Bioengineering, Changsha University of Science and Technology, Changsha 410076, People’s Republic of China

## Abstract

The title compound, C_19_H_13_BrClN, was synthesized by *N*-alkyl­ation of 4-chloro-1-(chloro­meth­yl)benzene with 3-bromo-9*H*-carbazole. The carbazole ring system is essentially planar, with a mean deviation of 0.028 Å, and it makes a dihedral angle of 91.2 (3) Å with the plane of the benzene ring.

## Related literature

For the pharmaceutical properties of the title compound, see: Buu-Hoï & Royer (1950 ▶); Caulfield *et al.* (2002 ▶); Harfenist & Joyner (1983 ▶); Harper *et al.* (2002 ▶). For bond-length data, see Allen *et al.* (1987 ▶). For synthetic procedures, see: Duan *et al.* (2005*a* ▶,*b* ▶).
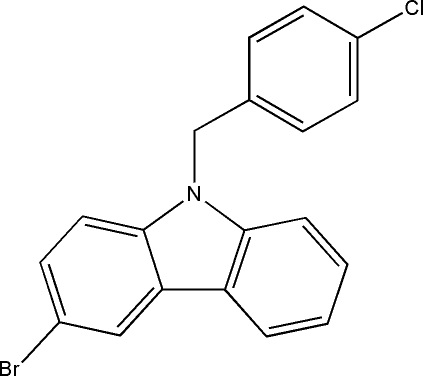


## Experimental

### 

#### Crystal data

C_19_H_13_BrClN
*M* *_r_* = 370.66
Orthorhombic, 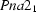

*a* = 17.272 (4) Å
*b* = 15.789 (3) Å
*c* = 5.5948 (11) Å
*V* = 1525.7 (5) Å^3^
*Z* = 4
Mo *K*α radiation
μ = 2.86 mm^−1^
*T* = 113 K
0.18 × 0.16 × 0.08 mm

#### Data collection

Rigaku Saturn CCD area-detector diffractometer
Absorption correction: multi-scan (*CrystalClear*; Rigaku, 2005 ▶) *T*_min_ = 0.627, *T*_max_ = 0.803
10796 measured reflections
2664 independent reflections
2401 reflections with *I* > 2σ(*I*)
*R*_int_ = 0.031

#### Refinement

*R*[*F* ^2^ > 2σ(*F* ^2^)] = 0.023
*wR*(*F* ^2^) = 0.056
*S* = 1.04
2664 reflections
199 parameters
1 restraint
H-atom parameters constrained
Δρ_max_ = 0.38 e Å^−3^
Δρ_min_ = −0.47 e Å^−3^
Absolute structure: Flack (1983 ▶), 1163 Friedel pairs
Flack parameter: 0.014 (9)

### 

Data collection: *CrystalClear* (Rigaku, 2005 ▶); cell refinement: *CrystalClear*; data reduction: *CrystalClear*; program(s) used to solve structure: *SHELXS97* (Sheldrick, 2008 ▶); program(s) used to refine structure: *SHELXL97* (Sheldrick, 2008 ▶); molecular graphics: *SHELXTL* (Sheldrick, 2008 ▶); software used to prepare material for publication: *SHELXL97*.

## Supplementary Material

Crystal structure: contains datablocks I, global. DOI: 10.1107/S1600536809019448/dn2457sup1.cif

Structure factors: contains datablocks I. DOI: 10.1107/S1600536809019448/dn2457Isup2.hkl

Additional supplementary materials:  crystallographic information; 3D view; checkCIF report

## Figures and Tables

**Fig. 1.**
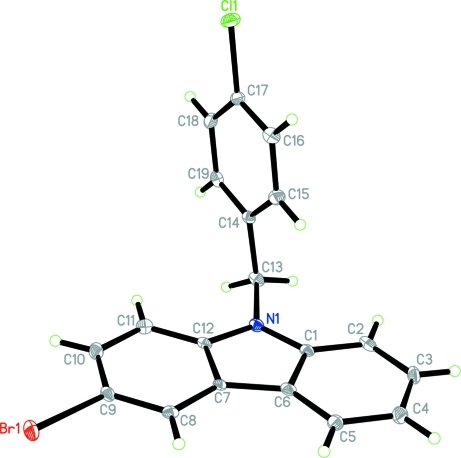
A view of the molecular structure of (I). Displacement ellipsoids are drawn at the 30% probability level (arbitrary spheres for H atoms).

## supplementary crystallographic information

### Comment

Carbazole derivatives substituted by *N*-alkylation possess valuable pharmaceutical properties (Buu-Hoï & Royer, 1950; Harfenist & Joyner, 1983; Caulfield *et al.*, 2002; Harper *et al.*, 2002). In this paper, the structure of 3-bromo-9-(4-chlorobenzyl)-9*H*-carbazole (I), synthesized by *N*-alkylation of 1-(chloromethyl)-4-chloroobenzene with 3-bromo-9*H*-carbazole, is reported

The carbazole ring is essentially planar, with mean deviations of 0.0275 Å. The dihedral angle between the carbazole ring and the benzyl ring is 91.2° A. The C—Br distance is 1.909 (3) Å, consistent with the literature (Allen *et al.*, 1987).

### Experimental

The title compound was prepared according to the procedure of Duan *et al.* (2005a,b). A solution of potassium hydroxide (0.67 g) in dimethylformamide (8 ml) was stirred at room temperature for 20 min. 3-Bromo-9*H*-carbazole (1.0 g, 4 mmol) was added and the mixture stirred for a further 40 min. A solution of 1-(chloromethyl)-4-chlorobenzene (0.97 g, 6 mmol) in dimethylformamide (5 ml) was added dropwise with stirring. The resulting mixture was then stirred at room temperature for 12 h and poured into water (100 ml), yielding a white precipitate. The solid product was filtered off, washed with cold water and recrystallized from EtOH, giving crystals of (I). Yield: 1.26 g (85.2%); m.p. 431 - 433 K. Compound (I) (40 mg) was dissolved in mixture of chloroform (5 ml) and ethanol (5 ml) and the solution was kept at room temperature for 14 d. Natural evaporation of the solution gave colourless crystals suitable for X-Ray analysis.

### Refinement

All H atoms were included in the riding model approximation with C—H distances = 0.93Å (benzene) and 0.97Å (methylene) with *U*_iso_(H)= 1.2x*U*_eq_(C).

### Crystal data

**Table:**

C_19_H_13_BrClN	*D*_x_ = 1.614 Mg m^−^^3^
*M**_r_* = 370.66	Melting point: 432 K
Orthorhombic, *P**n**a*2_1_	Mo *K*α radiation, λ = 0.71073 Å
Hall symbol: P 2c -2n	Cell parameters from 5012 reflections
*a* = 17.272 (4) Å	θ = 1.8–27.9°
*b* = 15.789 (3) Å	µ = 2.86 mm^−^^1^
*c* = 5.5948 (11) Å	*T* = 113 K
*V* = 1525.7 (5) Å^3^	Block, colorless
*Z* = 4	0.18 × 0.16 × 0.08 mm
*F*(000) = 744	

### Data collection

**Table:**

Rigaku Saturn CCD area-detector diffractometer	2664 independent reflections
Radiation source: rotating anode	2401 reflections with *I* > 2σ(*I*)
confocal	*R*_int_ = 0.031
Detector resolution: 7.31 pixels mm^-1^	θ_max_ = 25.0°, θ_min_ = 1.8°
ω and φ scans	*h* = −20→19
Absorption correction: multi-scan (*CrystalClear*; Rigaku, 2005)	*k* = −18→18
*T*_min_ = 0.627, *T*_max_ = 0.803	*l* = −6→6
10796 measured reflections	

### Refinement

**Table:**

Refinement on *F*^2^	Secondary atom site location: difference Fourier map
Least-squares matrix: full	Hydrogen site location: inferred from neighbouring sites
*R*[*F*^2^ > 2σ(*F*^2^)] = 0.023	H-atom parameters constrained
*wR*(*F*^2^) = 0.056	*w* = 1/[σ^2^(*F*_o_^2^) + (0.0295*P*)^2^] where *P* = (*F*_o_^2^ + 2*F*_c_^2^)/3
*S* = 1.04	(Δ/σ)_max_ = 0.001
2664 reflections	Δρ_max_ = 0.38 e Å^−^^3^
199 parameters	Δρ_min_ = −0.46 e Å^−^^3^
1 restraint	Absolute structure: Flack (1983), 1163 Friedel pairs
Primary atom site location: structure-invariant direct methods	Flack parameter: 0.014 (9)

### Special details

**Table:**

Geometry. All e.s.d.'s (except the e.s.d. in the dihedral angle between two l.s. planes) are estimated using the full covariance matrix. The cell e.s.d.'s are taken into account individually in the estimation of e.s.d.'s in distances, angles and torsion angles; correlations between e.s.d.'s in cell parameters are only used when they are defined by crystal symmetry. An approximate (isotropic) treatment of cell e.s.d.'s is used for estimating e.s.d.'s involving l.s. planes.
Refinement. Refinement of *F*^2^ against ALL reflections. The weighted *R*-factor *wR* and goodness of fit *S* are based on *F*^2^, conventional *R*-factors *R* are based on *F*, with *F* set to zero for negative *F*^2^. The threshold expression of *F*^2^ > σ(*F*^2^) is used only for calculating *R*-factors(gt) *etc*. and is not relevant to the choice of reflections for refinement. *R*-factors based on *F*^2^ are statistically about twice as large as those based on *F*, and *R*- factors based on ALL data will be even larger.

### Fractional atomic coordinates and isotropic or equivalent isotropic displacement parameters (Å^2^)

**Table:**

	*x*	*y*	*z*	*U*_iso_*/*U*_eq_	
Br1	0.258680 (12)	0.185476 (15)	1.32912 (10)	0.02288 (9)	
Cl1	0.65120 (4)	0.59416 (4)	0.8259 (2)	0.02847 (16)	
N1	0.53701 (13)	0.18736 (14)	0.6844 (4)	0.0167 (5)	
C1	0.58534 (13)	0.13162 (15)	0.8041 (6)	0.0152 (6)	
C2	0.66094 (15)	0.10636 (17)	0.7491 (5)	0.0209 (7)	
H2	0.6862	0.1270	0.6143	0.025*	
C3	0.69681 (15)	0.04979 (18)	0.9013 (5)	0.0224 (7)	
H3	0.7471	0.0322	0.8682	0.027*	
C4	0.65932 (16)	0.01831 (19)	1.1042 (6)	0.0233 (7)	
H4	0.6848	−0.0198	1.2038	0.028*	
C5	0.58446 (15)	0.04349 (18)	1.1579 (5)	0.0186 (7)	
H5	0.5597	0.0227	1.2934	0.022*	
C6	0.54667 (15)	0.10001 (16)	1.0080 (5)	0.0167 (6)	
C7	0.47140 (14)	0.13996 (16)	1.0130 (5)	0.0135 (6)	
C8	0.40812 (14)	0.13595 (18)	1.1675 (5)	0.0160 (6)	
H8	0.4082	0.1001	1.2993	0.019*	
C9	0.34553 (15)	0.18694 (17)	1.1181 (5)	0.0174 (6)	
C10	0.34293 (15)	0.24129 (18)	0.9223 (5)	0.0210 (7)	
H10	0.2993	0.2746	0.8960	0.025*	
C11	0.40475 (15)	0.24581 (18)	0.7676 (5)	0.0202 (7)	
H11	0.4040	0.2824	0.6373	0.024*	
C12	0.46844 (13)	0.19405 (14)	0.8117 (6)	0.0147 (5)	
C13	0.55872 (15)	0.24502 (16)	0.4941 (5)	0.0193 (7)	
H13A	0.5160	0.2495	0.3822	0.023*	
H13B	0.6026	0.2214	0.4087	0.023*	
C14	0.57980 (15)	0.33317 (17)	0.5812 (5)	0.0151 (6)	
C15	0.62228 (15)	0.34401 (18)	0.7921 (6)	0.0227 (7)	
H15	0.6358	0.2971	0.8835	0.027*	
C16	0.64432 (14)	0.42472 (17)	0.8658 (6)	0.0215 (7)	
H16	0.6729	0.4319	1.0052	0.026*	
C17	0.62343 (16)	0.49356 (17)	0.7311 (5)	0.0187 (6)	
C18	0.58085 (15)	0.48457 (18)	0.5212 (6)	0.0223 (7)	
H18	0.5671	0.5318	0.4313	0.027*	
C19	0.55924 (15)	0.40353 (17)	0.4486 (5)	0.0192 (7)	
H19	0.5306	0.3967	0.3091	0.023*	

### Atomic displacement parameters (Å^2^)

**Table:**

	*U*^11^	*U*^22^	*U*^33^	*U*^12^	*U*^13^	*U*^23^
Br1	0.01586 (13)	0.02907 (15)	0.02370 (15)	0.00028 (9)	0.0041 (2)	−0.0042 (2)
Cl1	0.0313 (3)	0.0172 (3)	0.0370 (4)	−0.0025 (2)	0.0062 (6)	−0.0066 (5)
N1	0.0162 (12)	0.0175 (14)	0.0164 (13)	−0.0024 (9)	0.0028 (10)	0.0012 (10)
C1	0.0157 (11)	0.0141 (13)	0.0159 (16)	−0.0043 (9)	−0.0006 (15)	−0.0046 (15)
C2	0.0167 (14)	0.0249 (17)	0.0210 (17)	−0.0076 (12)	0.0033 (11)	−0.0064 (12)
C3	0.0131 (14)	0.0229 (17)	0.031 (2)	0.0027 (11)	−0.0004 (12)	−0.0099 (13)
C4	0.0196 (16)	0.0219 (17)	0.0283 (18)	0.0007 (13)	−0.0074 (13)	−0.0015 (14)
C5	0.0187 (16)	0.0179 (16)	0.0193 (16)	−0.0019 (12)	−0.0037 (13)	−0.0021 (12)
C6	0.0161 (14)	0.0147 (16)	0.0192 (16)	−0.0050 (11)	−0.0006 (12)	−0.0059 (13)
C7	0.0121 (13)	0.0118 (15)	0.0166 (15)	−0.0036 (10)	−0.0011 (12)	−0.0024 (12)
C8	0.0184 (15)	0.0137 (16)	0.0159 (16)	−0.0045 (11)	−0.0051 (12)	−0.0018 (12)
C9	0.0128 (13)	0.0187 (16)	0.0208 (17)	−0.0032 (12)	0.0023 (12)	−0.0078 (13)
C10	0.0161 (15)	0.0222 (17)	0.0248 (17)	0.0011 (11)	−0.0055 (12)	−0.0029 (13)
C11	0.0232 (14)	0.0199 (15)	0.017 (2)	−0.0010 (11)	−0.0014 (12)	0.0006 (12)
C12	0.0155 (11)	0.0150 (13)	0.0137 (14)	−0.0051 (9)	0.0017 (18)	−0.0022 (16)
C13	0.0193 (15)	0.0235 (17)	0.0151 (15)	−0.0050 (12)	0.0030 (13)	−0.0026 (14)
C14	0.0138 (14)	0.0174 (15)	0.0142 (15)	−0.0024 (11)	0.0050 (12)	0.0002 (12)
C15	0.0235 (13)	0.0193 (14)	0.025 (2)	−0.0009 (10)	−0.0009 (16)	0.0049 (15)
C16	0.0220 (13)	0.0251 (16)	0.017 (2)	−0.0035 (10)	0.0012 (14)	−0.0007 (14)
C17	0.0181 (14)	0.0149 (16)	0.0229 (16)	−0.0008 (11)	0.0056 (12)	−0.0012 (12)
C18	0.0201 (15)	0.0194 (17)	0.0273 (18)	0.0039 (12)	0.0024 (14)	0.0017 (14)
C19	0.0145 (14)	0.0261 (18)	0.0172 (16)	0.0001 (11)	0.0007 (12)	0.0013 (13)

### Geometric parameters (Å, °)

**Table:**

Br1—C9	1.909 (3)	C8—H8	0.9300
Cl1—C17	1.742 (3)	C9—C10	1.392 (4)
N1—C1	1.385 (3)	C10—C11	1.376 (4)
N1—C12	1.386 (3)	C10—H10	0.9300
N1—C13	1.450 (3)	C11—C12	1.393 (4)
C1—C2	1.399 (3)	C11—H11	0.9300
C1—C6	1.413 (4)	C13—C14	1.519 (4)
C2—C3	1.381 (4)	C13—H13A	0.9700
C2—H2	0.9300	C13—H13B	0.9700
C3—C4	1.398 (4)	C14—C19	1.382 (4)
C3—H3	0.9300	C14—C15	1.400 (4)
C4—C5	1.386 (4)	C15—C16	1.392 (4)
C4—H4	0.9300	C15—H15	0.9300
C5—C6	1.388 (4)	C16—C17	1.371 (4)
C5—H5	0.9300	C16—H16	0.9300
C6—C7	1.445 (3)	C17—C18	1.393 (4)
C7—C8	1.395 (4)	C18—C19	1.393 (4)
C7—C12	1.415 (4)	C18—H18	0.9300
C8—C9	1.376 (4)	C19—H19	0.9300
			
C1—N1—C12	108.4 (2)	C9—C10—H10	119.9
C1—N1—C13	126.7 (2)	C10—C11—C12	118.1 (3)
C12—N1—C13	123.4 (2)	C10—C11—H11	121.0
N1—C1—C2	129.6 (3)	C12—C11—H11	121.0
N1—C1—C6	109.2 (2)	N1—C12—C11	129.0 (3)
C2—C1—C6	121.2 (3)	N1—C12—C7	109.4 (2)
C3—C2—C1	117.9 (3)	C11—C12—C7	121.6 (3)
C3—C2—H2	121.1	N1—C13—C14	113.7 (2)
C1—C2—H2	121.1	N1—C13—H13A	108.8
C2—C3—C4	121.5 (3)	C14—C13—H13A	108.8
C2—C3—H3	119.2	N1—C13—H13B	108.8
C4—C3—H3	119.2	C14—C13—H13B	108.8
C5—C4—C3	120.4 (3)	H13A—C13—H13B	107.7
C5—C4—H4	119.8	C19—C14—C15	119.3 (3)
C3—C4—H4	119.8	C19—C14—C13	120.2 (3)
C4—C5—C6	119.5 (3)	C15—C14—C13	120.5 (3)
C4—C5—H5	120.3	C16—C15—C14	120.3 (3)
C6—C5—H5	120.3	C16—C15—H15	119.8
C5—C6—C1	119.5 (2)	C14—C15—H15	119.8
C5—C6—C7	133.8 (3)	C17—C16—C15	119.4 (3)
C1—C6—C7	106.7 (2)	C17—C16—H16	120.3
C8—C7—C12	119.5 (2)	C15—C16—H16	120.3
C8—C7—C6	134.2 (3)	C16—C17—C18	121.4 (3)
C12—C7—C6	106.3 (2)	C16—C17—Cl1	118.9 (2)
C9—C8—C7	117.7 (3)	C18—C17—Cl1	119.7 (2)
C9—C8—H8	121.2	C17—C18—C19	118.8 (3)
C7—C8—H8	121.2	C17—C18—H18	120.6
C8—C9—C10	123.0 (3)	C19—C18—H18	120.6
C8—C9—Br1	119.1 (2)	C14—C19—C18	120.8 (3)
C10—C9—Br1	117.9 (2)	C14—C19—H19	119.6
C11—C10—C9	120.1 (3)	C18—C19—H19	119.6
C11—C10—H10	119.9		
			
C12—N1—C1—C2	−178.2 (3)	C9—C10—C11—C12	0.8 (4)
C13—N1—C1—C2	−12.0 (4)	C1—N1—C12—C11	176.2 (3)
C12—N1—C1—C6	1.6 (3)	C13—N1—C12—C11	9.5 (4)
C13—N1—C1—C6	167.8 (2)	C1—N1—C12—C7	−1.7 (3)
N1—C1—C2—C3	179.4 (3)	C13—N1—C12—C7	−168.4 (2)
C6—C1—C2—C3	−0.4 (4)	C10—C11—C12—N1	−179.6 (3)
C1—C2—C3—C4	0.1 (4)	C10—C11—C12—C7	−1.9 (4)
C2—C3—C4—C5	−0.1 (4)	C8—C7—C12—N1	−179.7 (2)
C3—C4—C5—C6	0.3 (4)	C6—C7—C12—N1	1.2 (3)
C4—C5—C6—C1	−0.6 (4)	C8—C7—C12—C11	2.2 (4)
C4—C5—C6—C7	−178.3 (3)	C6—C7—C12—C11	−177.0 (2)
N1—C1—C6—C5	−179.2 (2)	C1—N1—C13—C14	−93.3 (3)
C2—C1—C6—C5	0.6 (4)	C12—N1—C13—C14	70.8 (3)
N1—C1—C6—C7	−0.9 (3)	N1—C13—C14—C19	−142.8 (3)
C2—C1—C6—C7	178.9 (2)	N1—C13—C14—C15	39.3 (4)
C5—C6—C7—C8	−1.2 (5)	C19—C14—C15—C16	−0.7 (4)
C1—C6—C7—C8	−179.1 (3)	C13—C14—C15—C16	177.2 (2)
C5—C6—C7—C12	177.8 (3)	C14—C15—C16—C17	0.6 (4)
C1—C6—C7—C12	−0.1 (3)	C15—C16—C17—C18	−0.3 (4)
C12—C7—C8—C9	−1.3 (4)	C15—C16—C17—Cl1	179.8 (2)
C6—C7—C8—C9	177.6 (3)	C16—C17—C18—C19	0.1 (4)
C7—C8—C9—C10	0.2 (4)	Cl1—C17—C18—C19	180.0 (2)
C7—C8—C9—Br1	−178.51 (19)	C15—C14—C19—C18	0.6 (4)
C8—C9—C10—C11	0.0 (4)	C13—C14—C19—C18	−177.4 (2)
Br1—C9—C10—C11	178.8 (2)	C17—C18—C19—C14	−0.2 (4)

## References

[bb1] Allen, F. H., Kennard, O., Watson, D. G., Brammer, L., Orpen, A. G. & Taylor, R. (1987). *J. Chem. Soc. Perkin Trans. 2*, pp. S1–19.

[bb2] Buu-Hoï, N. P. & Royer, R. (1950). *J. Org. Chem.* **15**, 123–130.

[bb3] Caulfield, T., Cherrier, M. P., Combeau, C. & Mailliet, P. (2002). Eur. Patent EP 1253141.

[bb4] Duan, X. M., Han, J., Chen, L. G., Xu, Y. J. & Li, Y. (2005*a*). *Fine Chem.* **22**, 39–40.

[bb5] Duan, X. M., Han, J., Chen, L. G., Xu, Y. J. & Li, Y. (2005*b*). *Fine Chem.* **22**, 52.

[bb6] Flack, H. D. (1983). *Acta Cryst.* A**39**, 876–881.

[bb7] Harfenist, M. & Joyner, C. T. (1983). US Patent No. 4 379 160.

[bb8] Harper, R. W., Lin, H. S. & Richett, M. E. (2002). World Patent WO2002079154.

[bb9] Rigaku (2005). *CrystalClear* Rigaku Americas Corporation, The Woodlands, Texas, USA.

[bb10] Sheldrick, G. M. (2008). *Acta Cryst.* A**64**, 112–122. 10.1107/S0108767307043930 18156677

